# Thanking cell & bioscience reviewers

**DOI:** 10.1186/2045-3701-5-6

**Published:** 2015-01-30

**Authors:** Yun-Bo Shi

**Affiliations:** Cell & Bioscience, Kragujevac, USA

## Abstract

The editors of *Cell & Bioscience* would like to thank all the reviewers who contributed to the journal in 2014.

**Sunil Acharya**

United States of America

**Jordi Barbe**

Spain

**Vsevolod Belousov**

Russian Federation

**Beatrice Cardinali**

Italy

**Petr Cejka**

Switzerland

**Pooja Chadha**

United States of America

**Wai Yee Chan**

China

**Siew Wee Chan**

Singapore

**Vera SF Chan**

Hong Kong

**Chuang-Rung Chang**

Taiwan

**Raymond Chuen-Chung Chang**

Hong Kong

**Yu-sun Chang**

Taiwan

**Bo-Shiun Chen**

United States of America

**Chaoping Chen**

United States of America

**Yonglong Chen**

China

**Didi Chen**

United States of America

**Guoxun Chen**

United States of America

**Pei-Yu Chen**

United States of America

**Rong Chen**

Australia

**Wei-June Chen**

Taiwan

**Xiaolei Chen**

United States of America

**Yu Chen**

United States of America

**Ya-Hui Chi**

Taiwan

**Justin Jang Hann Chu**

Singapore

**Mingxing Chu**

China

**Eva Corey**

United States of America

**Mary Dasso**

United States of America

**Lester Davids**

South Africa

**Matthew DeBerge**

United States of America

**Changwang Deng**

United States of America

**Qiurong Ding**

United States of America

**Sheng Ding**

United States of America

**Jingqi Duan**

United States of America

**Elia Duh**

United States of America

**Qian Feng**

Netherlands

**Yulong Fu**

United States of America

**Akiyoshi Fukamizu**

Japan

**Qiang Gan**

United States of America

**Guoquan Gao**

China

**Kai Ge**

United States of America

**Ai-Di Gu**

United States of America

**Peixuan Guo**

United States of America

**Sanjay Gupta**

United States of America

**Brett Hambly**

Australia

**Emma Hamilton-Williams**

Australia

**Zhipeng Han**

China

**Lawrence Hayward**

United States of America

**Liqiang He**

United States of America

**Yunlong He**

United States of America

**Shanshan He**

United States of America

**Yunlong He**

United States of America

**Victor Hsia**

United States of America

**Yi-Chiang Hsu**

Taiwan

**Jiancheng Hu**

United States of America

**Wenhui Hu**

United States of America

**Chuanshu Huang**

United States of America

**Jian Huang**

China

**Malik Hussain**

Australia

**Yoshifumi Itoh**

United Kingdom

**Zongchao Jia**

Canada

**Lili Jiang**

China

**Sibo Jiang**

United States of America

**Peng Jin**

United States of America

**Shouguang Jin**

United States of America

**Hung-Ying Kao**

United States of America

**James L. Keck**

United States of America

**Tatsuo Kido**

United States of America

**Hwan Myung Kim**

Korea

**Chi-Hon Lee**

United States of America

**Tae Jin Lee**

United States of America

**Sy-Jye Leu**

Taiwan

**Chuan-Yun Li**

China

**Qihan Li**

China

**Chao Li**

United States of America

**Mingtao Li**

China

**Zichong Li**

United States of America

**Qiao Li**

Canada

**Shengwen Calvin Li**

United States of America

**Xuhang Li**

United States of America

**Zhongji Liao**

United States of America

**Aifu Lin**

United States of America

**Ding-Yen Lin**

Taiwan

**Xiaowen Liu**

United States of America

**Shan-Lu Liu**

United States of America

**Kiatlyn Liu**

United States of America

**Amy Lo**

Hong Kong

**Jianfeng Lu**

United States of America

**Zhigang Lu**

United States of America

**Vivian Lui**

Hong Kong

**Li Ma**

United States of America

**M. Murali Kannan Maruthamuthu**

India

**Craig McLachlan**

Australia

**Duojiao Ni**

United States of America

**Richard Niles**

United States of America

**Suman Paul**

United States of America

**Aimin Peng**

United States of America

**Xiao Peng**

United States of America

**Harish Nair Ramanathan**

United States of America

**G V Rao**

India

**Ursula Rauch**

Germany

**William Redmond**

United States of America

**Jelena Rnjak-Kovacina**

Australia

**Jean-Marc Sabatier**

France

**Mitali Sarkar-Tyson**

United Kingdom

**Kristina Schmidt**

United States of America

**David Schneider**

United States of America

**Huanjie Shao**

United States of America

**Ashwani Sharma**

United States of America

**Qunxin She**

Denmark

**C. K. James Shen**

Taiwan

**Han-Ming Shen**

Singapore

**Jinfeng Shen**

United States of America

**Bing Shen**

China

**Xuan-Zheng Shi**

United States of America

**Yufang Shi**

China

**Sanjeev Shukla**

United States of America

**Jay Singh**

United States of America

**Wenqiang Song**

United States of America

**Yang Song**

United States of America

**Marina Stanilova**

Bulgaria

**Yi Henry Sun**

Taiwan

**Kai Sun**

China

**Mahima Swamy**

United Kingdom

**Dorian Swarts**

United Kingdom

**Katalin Szaszi**

Canada

**Ming Tan**

United States of America

**Yuichiro Tanaka**

United States of America

**Sai-Wen Tang**

United States of America

**Yanmei Tao**

China

**Chenxi Tian**

United States of America

**Ming-Jer Tsai**

United States of America

**Toshikazu Ushijima**

Japan

**Alessandro Vindigni**

United States of America

**Bing Wang**

United States of America

**Honghe Wang**

United States of America

**Rongfu Wang**

United States of America

**Shaoying Wang**

United States of America

**Tuanlao Wang**

China

**Xingsheng Wang**

United States of America

**Yi-Ching Wang**

Taiwan

**Yu Wang**

United States of America

**Lixin Wei**

China

**Qing Wei**

United States of America

**Tao Weitao**

United States of America

**Jiemin Wong**

China

**Chi-Ming Wong**

Hong Kong

**Min Wu**

United States of America

**Gutian Xiao**

United States of America

**Ping Xie**

China

**Shunbin Xiong**

United States of America

**Mingxuan Xu**

United States of America

**Li Ye**

United States of America

**Venkat Yedavalli**

United States of America

**Paul Yen**

Singapore

**Hao Ying**

China

**Kenji Yoshida**

Japan

**Chundong Yu**

China

**Dihua Yu**

United States of America

**Hanna Yuan**

Taiwan

**Ann Marie Zavacki**

United States of America

**Mu-Sheng Zeng**

China

**Jiangwei Zhang**

United States of America

**Mingliang Zhang**

United States of America

**Xiang Zhang**

United States of America

**Yanling Zhang**

United States of America

**Gong Zhang**

China

**Li Zhang**

China

**Zhengmao Zhang**

United States of America

**Richard Y Zhao**

United States of America

**Hui Zhao**

Hong Kong

**Zhengyi Zhao**

United States of America

**Chao Zheng**

United States of America

**Zhi-Ming Zheng**

United States of America

**Weimin Zhong**

United States of America

**Jun Zhou**

China

**Rongjia Zhou**

China

**Wen Zhou**

United States of America

**Zhou Zhou**

United States of America

**Jun Zhu**

United States of America

**Fangfang Zhu**

United States of America

**Shunyi Zhu**

China

**Wei-Xing Zong**

United States of America

